# Medical-Grade Silicone Coated with Rhamnolipid R89 Is Effective against *Staphylococcus* spp. Biofilms

**DOI:** 10.3390/molecules24213843

**Published:** 2019-10-25

**Authors:** Chiara Ceresa, Francesco Tessarolo, Devid Maniglio, Erica Tambone, Irene Carmagnola, Emanuele Fedeli, Iole Caola, Giandomenico Nollo, Valeria Chiono, Gianna Allegrone, Maurizio Rinaldi, Letizia Fracchia

**Affiliations:** 1 Department of Pharmaceutical Sciences, Università del Piemonte Orientale “A. Avogadro”, 28100 Novara, Italy; chiara.ceresa@uniupo.it (C.C.); erica.tambone@unitn.it (E.T.); emanuele.fedeli@uniupo.it (E.F.); gianna.allegrone@uniupo.it (G.A.); maurizio.rinaldi@uniupo.it (M.R.); 2 BIOtech Center for Biomedical Technologies, Department of Industrial Engineering, Università di Trento, 38123 Trento, Italy; francesco.tessarolo@unitn.it (F.T.); devid.maniglio@unitn.it (D.M.); giandomenico.nollo@unitn.it (G.N.); 3 Healthcare Research and Innovation Program (IRCS-FBK-PAT), Bruno Kessler Foundation, 38123 Trento, Italy; 4 Department of Mechanical and Aerospace Engineering, Politecnico di Torino, 10129 Turin, Italy; irene.carmagnola@polito.it (I.C.); valeria.chiono@polito.it (V.C.); 5 Section of Electron Microscopy, Department of Medicine Laboratory, Azienda Provinciale per i Servizi Sanitari di Trento, 38123 Trento, Italy; iole.caola@gmail.com

**Keywords:** biofilm, biosurfactants, *Staphylococcus* spp., antimicrobial, anti-adhesive, anti-biofilm, biomaterials, silicone, medical devices

## Abstract

*Staphylococcus aureus* and *Staphylococcus epidermidis* are considered two of the most important pathogens, and their biofilms frequently cause device-associated infections. Microbial biosurfactants recently emerged as a new generation of anti-adhesive and anti-biofilm agents for coating implantable devices to preserve biocompatibility. In this study, R89 biosurfactant (R89BS) was evaluated as an anti-biofilm coating on medical-grade silicone. R89BS is composed of homologues of the mono- (75%) and di-rhamnolipid (25%) families, as evidenced by mass spectrometry analysis. The antimicrobial activity against *Staphylococcus* spp. planktonic and sessile cells was evaluated by microdilution and metabolic activity assays. R89BS inhibited *S. aureus* and *S. epidermidis* growth with minimal inhibitory concentrations (MIC_99_) of 0.06 and 0.12 mg/mL, respectively and dispersed their pre-formed biofilms up to 93%. Silicone elastomeric discs (SEDs) coated by R89BS simple adsorption significantly counteracted *Staphylococcus* spp. biofilm formation, in terms of both built-up biomass (up to 60% inhibition at 72 h) and cell metabolic activity (up to 68% inhibition at 72 h). SEM analysis revealed significant inhibition of the amount of biofilm-covered surface. No cytotoxic effect on eukaryotic cells was detected at concentrations up to 0.2 mg/mL. R89BS-coated SEDs satisfy biocompatibility requirements for leaching products. Results indicate that rhamnolipid coatings are effective anti-biofilm treatments and represent a promising strategy for the prevention of infection associated with implantable devices.

## 1. Introduction

Given their effectiveness in improving patient health and quality of life, the use of implantable medical devices significantly increased over the years. Unfortunately, they can also act as a substrate for microbial colonization and biofilm development. In particular, approximately 60–70% of nosocomial infections are due to the development of microbial biofilms at the interface between the implanted device and tissues. [1]. Biofilms are defined as microbial communities derived from cells irreversibly attached to one another or to a substrate, embedded in a matrix of extracellular polymeric substances [2]. In this “lifestyle”, bacteria have slower metabolism, are more resistant to the host’s immune system, and are more tolerant to drug therapy than their planktonic counterparts [3,4].

*Staphylococcus aureus* and *Staphylococcus epidermidis*, due to their ability to form biofilms, are considered two of the most relevant pathogens in device-associated infections [5], affecting mainly hospitalized patients, immunocompromised or immunosuppressed subjects, and premature babies [6,7]. These Gram-positive bacteria are mainly introduced into the body during the surgical implantation of the device, thereby increasing the risk of surgical site contamination and implant colonization [5,8]. Implant colonization and subsequent biofilm formation often lead to the loss of device functionality and may represent a way to colonize new suitable sites. Surgical removal and replacement of the infected device is the most effective eradication strategy. However, this is not always applicable, and suppressive long-term multi-drug antibiotic therapies should be administered [9,10].

Several surface-treated biomaterials and medical devices with anti-biofilm properties were proposed [11,12,13,14,15,16,17,18,19], but most of them showed either potential toxicity to the peri-implant tissue cells or limited efficacy in clinical practice over time [20]. Therefore, new safe and effective approaches to prevent and treat biofilm infections are urgently required. The development of biocompatible coatings with antibacterial and/or anti-adhesive molecules represents one of the most promising approaches to reduce the incidence of implant-related infections [21].

Microbial biosurfactants (BSs) recently emerged as a new generation of “green” anti-biofilm and antimicrobial agents with enhanced biocompatibility [22,23,24,25,26,27].

Among microbial BSs, rhamnolipids from *Pseudomonas aeruginosa* showed relevant anti-biofilm potential in the medical field thanks to their antibacterial, anti-adhesive, and anti-biofilm properties [28,29,30].

The present study aimed at evaluating the antimicrobial activity and anti-biofilm properties of rhamnolipid R89, from *P. aeruginosa* 89. The antibacterial properties of the biosurfactant against planktonic cells of *S. aureus* and *S. epidermidis* were studied, as well as its effect on biofilm dispersal. Experiments on medical-grade silicone coated with R89BS were conducted against intermediate and mature staphylococcal biofilms. Results are discussed taking into consideration the R89BS chemistry, the physicochemical characteristics of the R89BS-coated surfaces, and the cytotoxicity of the biosurfactant and of the R89BS-coated silicone discs.

## 2. Results

### 2.1. Chemical Characterization of R89BS

**Figure 1:**
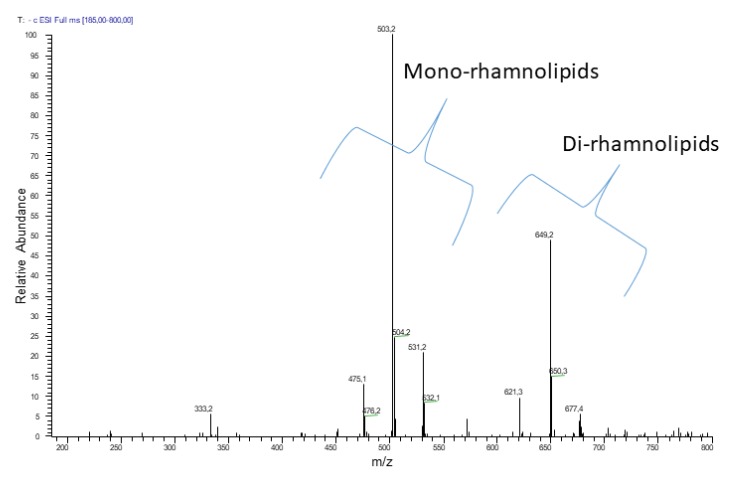
Negative electrospray ionization ((−)ESI) MS analysis (direct infusion) of rhamnolipids produced by *Pseudomonas aeruginosa* 89. Two clusters of peaks revealed two sets of homologue molecules. The first set evidenced three main signals corresponding to the [M − H]^−^ of mono-rhamnolipids. The second set evidenced three main signals corresponding to the deprotonated molecules of di-rhamnolipids.

The negative electrospray ionization (ESI) MS analysis of the crude biosurfactant R89BS extract showed the presence of homologues of mono- and di-rhamnolipids (Figure 1). The mono-rhamnolipid family members were composed mainly of C_10_–C_10_, C_8_–C_10_, and C_10_–C_12_, homologues, whose structures were confirmed by the product ion spectra of the precursor molecules [M − H]^−^ at *m/z* 503, 475, and 531 respectively. The di-rhamnolipid family members were represented by three main homologues corresponding to C_10_–C_8_, C_10_–C_10_, and C_10_–C_12_ homologues, whose structures were confirmed by the product ion spectra of the deprotonated molecules [M − H]^−^ at *m/z* 621, 649, and 677, respectively.

The relative amounts of the two groups in the crude extract were about 75% mono- and 25% di-rhamnolipids.

### 2.2. Antimicrobial Tests on Planktonic Cells

R89BS was checked as an antimicrobial agent on *Staphylococcus* spp. via the microdilution method. The effect of the crude R89 biosurfactant on the growth of planktonic cells of *S. aureus* ATCC^®^ 6538^TM^ and *S. epidermidis* ATCC^®^ 35984^TM^ was similar. The results showed an antibacterial activity of R89BS against both Gram-positive strains with minimal inhibitory concentration (MIC) values of 0.06 mg/mL and 0.12 mg/mL for *S. aureus* and *S. epidermidis*, respectively. The optical density values measured at 595 nm for the different R89BS concentrations are reported in Table 1. The corresponding percentages of inhibition, starting from the MICs determined visually, were ≥99.5%.

### 2.3. Disruption of Pre-Formed Biofilms

**Figure 2:**
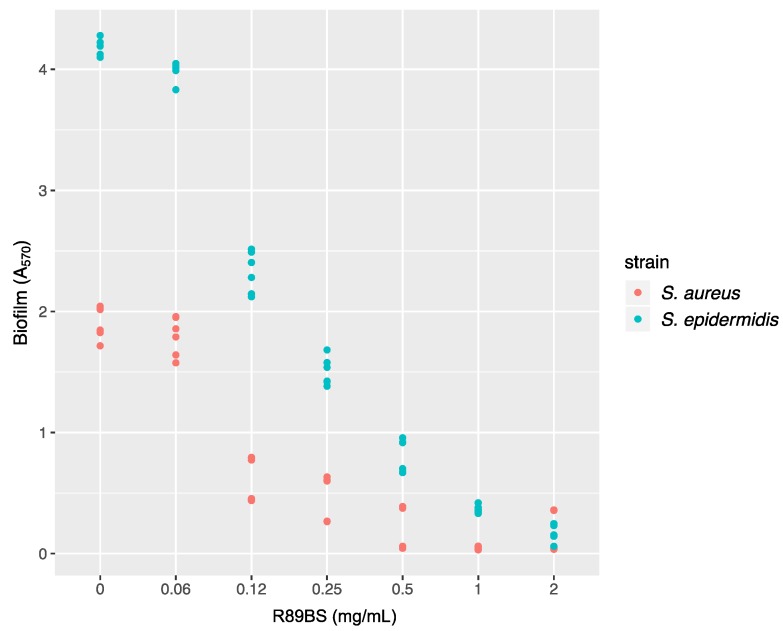
*Staphylococcus* spp. biofilm disruption by R89 biosurfactant (R89BS). *S. aureus* and *S. epidermidis* pre-formed biofilms were co-incubated with R89BS at concentrations ranging from 0.06 to 2 mg/mL. The disrupting activity of R89BS on *Staphylococcus* spp. biofilms was evaluated after 24 h at 37 °C. Dots (six for each concentration) represent single experimental data.

The ability of R89BS crude extract to influence the growth of *Staphylococcus* spp. pre-existing biofilms was determined after 24 h of co-incubation. Absorbance at 570 nm versus R89BS concentration is shown in Figure 2. R89BS significantly disrupted pre-formed biofilms of both *Staphylococcus* strains in a concentration-dependent manner (*p* < 1 × 10^−15^). In particular, as confirmed by Tukey post hoc test, pre-formed biofilms were significantly dislodged by R89BS concentrations from 0.12 to 2 mg/mL with biofilm reduction ranges of 68–89% for *S. aureus* and of 44–96% for *S. epidermidis* (*p* < 1 × 10^−4^). Conversely, no significant biofilm disruption activity was detected for R89BS at the concentration of 0.06 mg/mL (*p* > 5 × 10^−2^).

### 2.4. Surface Physicochemical Characterization

Surface physicochemical characterization was carried out on control silicone elastomeric discs (SEDs) (immersed in phosphate-buffered saline (PBS) only) and on SED samples incubated in R89BS solution at a concentration of 2 mg/mL.

SED samples showed a hydrophobic behavior with an advancing contact angle of 112° ± 5° and a receding angle of 81.0° ± 3.2°. The wettability increased significantly on samples coated with R89BS, settling on values of 84.4° ± 2.2° (advancing) and 72.2° ± 2.5° (receding), due to the introduction of hydrophilic groups, such as –OH, –COOH, and –NH_2_ present in R89BS molecules. It is worth noting that contact angle hysteresis in this case was quite small (about 12°), indicating a quite homogeneous distribution of the surfactant on the surface.

**Figure 3:**
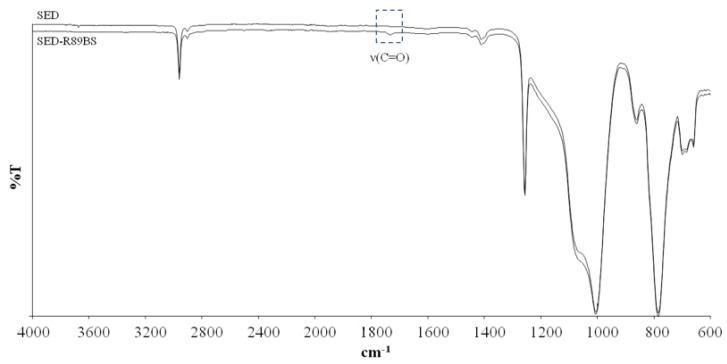
ATR-FTIR spectra of silicone elastomeric discs (SEDs) and SEDs coated with R89BS.

ATR-FTIR analysis was performed to study changes in the compositional properties at the surface of SED samples after R89BS adsorption. Figure 3 compares spectra of uncoated and R89BS-coated SED samples. The SED spectrum showed characteristic bands at 800–1110 cm^−1^ due to Si–O–Si stretching, at 1258 cm^−1^ due to CH_3_ symmetric stretching of Si–CH_3_, at 1412 cm^−1^ due to CH_3_ asymmetric stretching of Si–CH_3_, and at 2962.3 cm^−1^ due to C–H stretching [31]. After R89BS adsorption, a band appeared at 1735 cm^−1^, attributable to carbonyl (C=O) stretching, which is characteristic of the ester functional group of the rhamnolipid.

### 2.5. Anti-Biofilm Assay

#### 2.5.1. Biofilm Quantification

**Figure 4:**
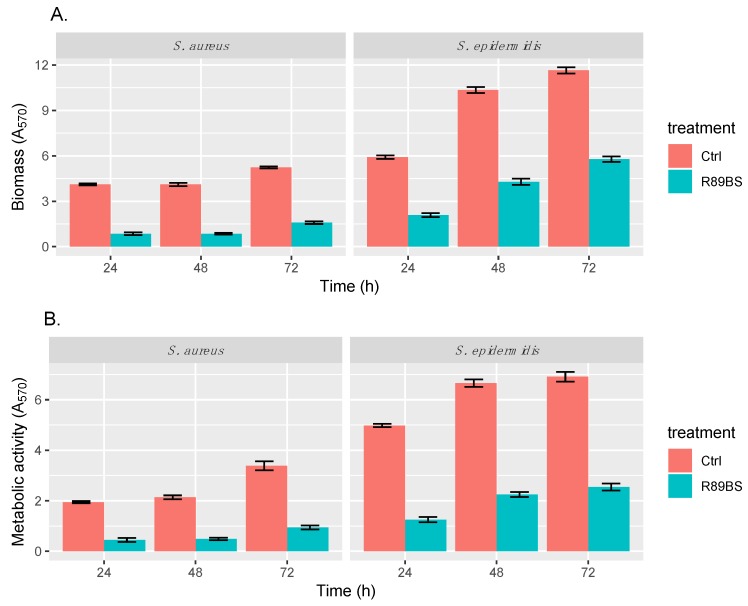
Anti-biofilm activity of R89BS deposited on the surface of silicone discs (SEDs) by direct physical adsorption. The anti-biofilm activity of R89BS-coated SEDs on *Staphylococcus* spp. biofilm formation was evaluated in terms of both biofilm biomass (**A**) and cell metabolic activity (**B**) after 24, 48, and 72 h.

The anti-biofilm activity of R89BS-coated SEDs against biofilm growth of *Staphylococcus* spp. on SEDs was detected up to 72 h of biofilm development (Figure 4). *S. aureus* biofilm was equally inhibited in terms of total biomass (Figure 4A) and cell metabolic activity (Figure 4B). For *S. epidermidis*, the biofilm was mostly reduced in terms of the metabolic activity of microbial cells (Figure 4B) rather than in terms of total biomass (Figure 4A).

For both strains, two-way ANOVA confirmed that biofilm biomass and metabolic activity were significantly dependent on the SED treatment (*p* < 1 × 10^−15^) and incubation time (*p* < 1 × 10^−15^). The efficacy of R89BS-coated SEDs against *S. aureus* biofilm formation was stable up to 48 h and decreased in part only at 72 h. In the case of *S. epidermidis*, the anti-biofilm activity of the R89BS coating reduced over time. Table 2 summarizes the 95% confidence intervals and *p*-values obtained for the tested *Staphylococcus* spp. in terms of both total biofilm biomass and cell metabolic activity. Overall, the pre-treatment of silicone surfaces with R89BS resulted in a biofilm inhibition of 76% for *S. aureus* and of 63% for *S. epidermidis* with respect to uncoated control SEDS. Details of percentages of biomass and metabolic activity inhibitions at the different time points are indicated in Table 3.

In order to confirm that R89BS activity was due to an anti-biofilm and not antibacterial action, the number of live cells was checked in the supernatant. For each treated and untreated SED, after 24, 48, and 72 h, growth media and washing solutions were collected. The presence of live bacteria was evaluated by cell metabolic assay (MTT method). The absorbance values corresponding to the supernatants of R89BS-coated SEDs were significantly higher in comparison to the obtained values for the supernatants of control SEDs (*p* < 0.01) suggesting that, in the wells containing the treated discs, vital cells lived in a planktonic state rather than forming a biofilm (Table 4).

#### 2.5.2. SEM Analysis of Bacterial Biofilms

**Figure 5:**
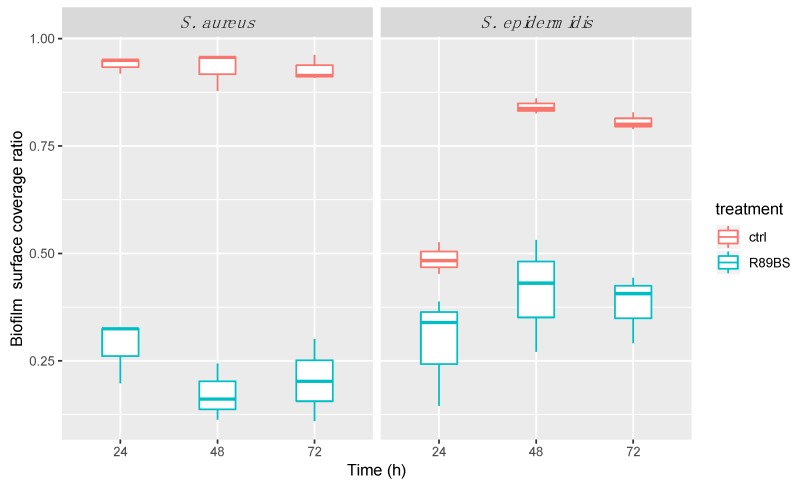
Quantitative evaluation of the biofilm surface coverage. Biofilm coverage surface was obtained by the analysis of a set of low-magnification SEM images obtained collecting a backscattered electron signal over a total surface of 29 mm^2^ at the SED center. R89BS-coated SEDs showed lower coverage values with respect to control SEDs, irrespectively of the incubation time points (24 h, 48 h, 72 h) for both the tested *Staphylococcus* spp.

SEM observation of the SED surface evidenced a marked difference in the amount, distribution, and microstructure of the adhering microbial biofilm. Results of the quantitative analysis showed that the *S. aureus* biofilm covered most of the surface of control SEDs within 24 h. A similar feature was obtained for *S. epidermidis* within 48 h. Most importantly, the biofilm surface coverage was significantly lower in R89BS-coated SEDs at all the considered time points. Quantitative results are summarized in Figure 5.

**Figure 6:**
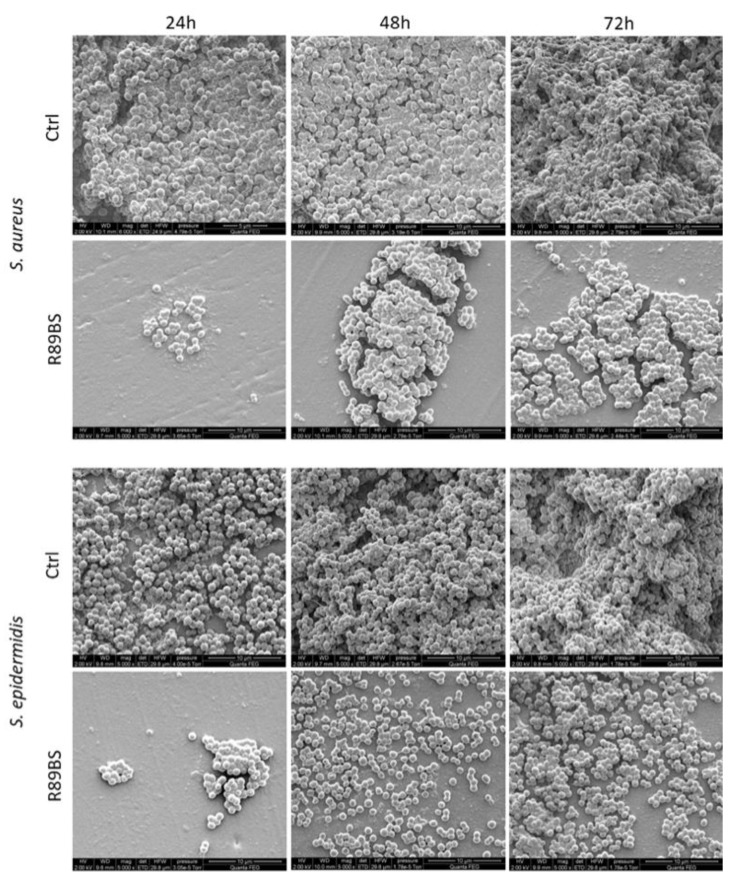
Micromorphology of the biofilm on the SED surface. Coccoid microbial cells and an extracellular matrix were present at the SED surface in different amounts according to incubation time (24 h, 48 h, and 72 h) and type of sample (Ctrl: uncoated controls; R89BS: coated silicone discs). A three-dimensional architecture was also revealed in control samples at incubation times longer than 24 h. Images were captured using scanning electron microscopy, high vacuum mode (primary electron beam energy, 2 keV; original magnification 5000×).

Qualitative analysis at higher magnification confirmed that the *S. aureus* biofilm appeared more structured than that of *S. epidermidis* at 24 h in control SEDs. However, a three-dimensional architecture and extracellular matrix were present in all control samples (untreated SEDs) at 48 h and 72 h of incubation. A marked three-dimensional architecture typical of a mature biofilm was evident for both *S. aureus* and *S. epidermidis* at 72 h on uncoated silicone discs. On the contrary, small and isolated aggregates of bacteria were identified at 24 h in R89BS samples for both strains. More structured aggregates were documented for *S. aureus* on treated SEDs at 48 h, showing elongated biofilm super-structures that increased in size at 72 h. In contrast, *S. epidermidis* on treated SEDs showed a more uniform cell distribution, with an increasing cell density at higher incubation times. The three-dimensional development of biofilms for both *Staphylococcus* strains was very limited on R89BS-coated surfaces. Typical features of a mature biofilm were identified only in limited portions of the R89BS-coated surfaces at 72 h of incubation. A representative selection of SEM images showing biofilm micromorphology is reported in Figure 6.

### 2.6. Cytotoxicity Analysis

**Figure 7:**
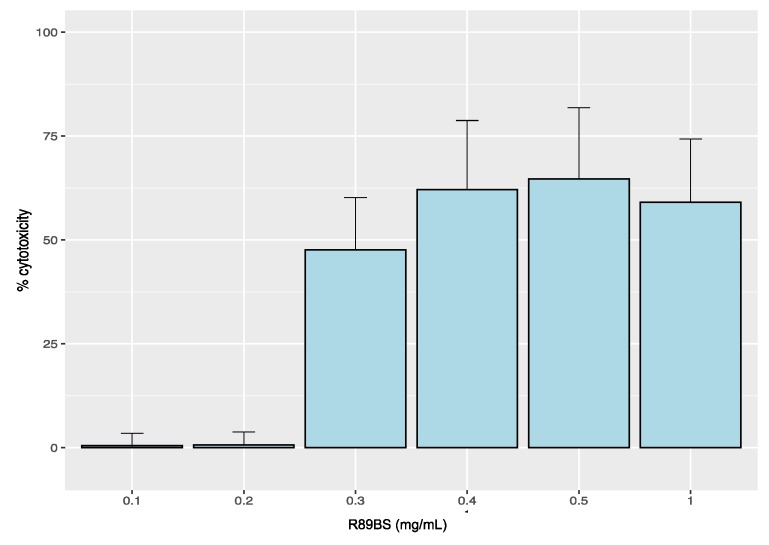
Cytotoxicity of R89BS on human normal lung fibroblasts (MRC5). Cytotoxicity level of different concentrations of R89BS solution. The negative control is represented by cells in standard growth medium, and the positive control is represented by fully lysate cells (0.5% Triton X).

Results from the R89BS cytotoxicity test on an MRC5 human normal lung fibroblast cell line are shown in Figure 7. A cytotoxic effect (viability from 41% to 64%) was documented for direct exposure to R89BS concentrations from 0.3 mg/mL to 1 mg/mL (*p* > 0.05 in comparison with positive control). On the contrary, no significant cytotoxicity was found for concentrations less than or equal to 0.2 mg/mL.

Furthermore, no cytotoxic effect was detected for the coated SED eluates. MRC5 cell viability after 48 h of exposure to the eluates obtained from both static and dynamic release conditions was comparable to negative controls.

## 3. Discussion

The extensive use of medical devices improved patient quality of life but also exposed them to an even higher risk of infection related to biofilm formation on these materials [22,32]. Bacteria attachment on the surfaces of medical devices is the basis for serious healthcare-associated infections (HCAI) correlated with significant morbidity and mortality [10]. In particular, *S. aureus* and *S. epidermidis* are recognized as the most frequent causes of medical device-associated infections (DRIs) due to their numerous virulence factors (e.g., production of toxins and biofilm formation) and their ability to evade immune defenses [33].

New strategies to prevent biofilm formation or promote their dispersal via cell removal or killing are, therefore, urgently required. Biosurfactants represent a new generation of anti-biofilm agents with potential applicability in the pharmaceutical and biomedical fields [23,34].

These amphiphilic molecules, produced by several microorganisms as secondary metabolites, are characterized by the presence of a hydrophilic and a hydrophobic portion, providing a peculiar surface activity at the interface [35,36]. They can provide direct biocidal action against several microorganisms by destabilizing cell membranes, altering their integrity and permeability [37,38,39]. Furthermore, BSs also indirectly interfere with cell adhesion and, consequently, with biofilm formation by modulating microbial interaction with surfaces [40,41]. The advantages of biosurfactants over their chemical counterpart are their specific action, low toxicity, high biodegradability, good performance in extreme environmental conditions, and unique structures [24,42]. In particular, in the last few years, the interest of the scientific community focused on rhamnolipids produced by *P. aeruginosa* [43,44,45]. In the present study, we provide evidence on the antimicrobial activity of the rhamnolipid biosurfactant produced by *P. aeruginosa* 89 against *S. aureus* ATCC^®^ 6538^TM^ and *S. epidermidis* ATCC^®^ 35984^TM^ and on R89BS anti-biofilm activity, in terms of both biofilm formation inhibition and biofilm dispersal. In addition, the chemical composition of R89BS, the physicochemical characteristics of coated surfaces, and cytotoxicity assays are presented.

The experimental setting was designed following a clinically oriented approach, using microbial species and a coated substrate, which are relevant in the biomedical context. For both staphylococcal strains, culture media and experimental conditions were chosen in order to promote their growth and the development of structured biofilms. To mimic and test a simple medical device having anti-biofilm properties, a rhamnolipid coating was realized on medical-grade silicone elastomer discs using a pre-coating method based on R89BS physical adsorption at the silicone surface. A number of complementary aspects of biofilm such as biomass, cell metabolic activity, biofilm disc coverage, and micro-structural characterization were considered to characterize and understand the coating anti-biofilm effect.

There are two important strategies for potentially reducing early infections of implantable devices: the reduction of the number of cells able to adhere to the surface and the delay of adhesion. R89BS is able to satisfy both these needs through its antimicrobial and anti-adhesive activities.

Firstly, the antibacterial activity of R89BS against both planktonic and sessile cells was taken into account in order to evaluate a possible use of this compound as an adjuvant of antibiotic treatment, being active at non-cytotoxic concentrations. Previous studies showed, in fact, that treatments with biosurfactants may facilitate the efficacy of combined antimicrobial drug therapies, which in most cases are less efficient against biofilms [46,47,48,49].

When dispersed in a solution, R89BS showed antibacterial activity against staphylococcal planktonic cells and promoted the effective removal of biofilms grown on SEDs. These results are in agreement with previous literature findings on others rhamnolipid biosurfactants. Ndlovu et al. [25] detected the pronounced antimicrobial activity of a rhamnolipid from *P. aeruginosa* ST5 against a broad spectrum of opportunistic and pathogenic microorganisms by means of the agar disc susceptibility test. Lotfabad et al. [26] observed a remarkable inhibitory effect of rhamnolipid mixtures (MR01 and MASH1) produced by *P. aeruginosa* strains against Gram-positive bacteria with MIC values equal to R89BS and comparable to antibiotics, posing them as a promising alternative to chemical drugs in the treatment of bacterial infections. Similar results were also reported for some *P. aeruginosa* rhamnolipid derivatives, which exhibited bactericidal activity against *S. aureus* ATCC 25923 and an *S. aureus* MRSA strain with an MIC of 62.5 μg/mL, as well as an interesting biofilm dispersion activity [50].

In addition, and most relevant for implant applications, R89BS was shown to be an effective and long-lasting anti-adhesive/anti-biofilm formation agent when used for coating medical-grade silicone. To our knowledge, this is the first time that the anti-biofilm activity of a rhamnolipid-based coating was evaluated on a silicone surface, obtaining significant anti-biofilm activities for incubation times up to three days. Other studies showed that a prior adsorption of rhamnolipids on surfaces can reduce bacterial adhesion and colonization [51,52,53,54]. De Araujo et al. [55] evaluated the potential of crude and purified rhamnolipid produced by *P. aeruginosa* PA1 on controlling *L. monocytogenes* and *P. fluorescens* biofilm formation with inhibitions up to 74% and 72% on polystyrene and on stainless-steel surfaces, respectively. Rodrigues et al. [27] verified the efficacy of rhamnolipids in reducing the initial deposition rate and the number of bacterial cells on voice prostheses after 4 h. In particular, a reduction in the adhesion rate of 66% for *S. salivarius* and *C. tropicalis* and a reduction in the number of adhering cells of 48% on biosurfactant-conditioned silicone for *S. epidermidis*, *S. salivarius*, *S. aureus*, and *C. tropicalis* were observed.

This study highlighted the potential of R89BS in preventing Gram-positive colonization of silicone implantable devices. The anti-biofilm action of the R89BS coating can be explained by the biosurfactant’s ability to modify the physicochemical properties of the silicone surface, increasing the surface hydrophilicity and preventing the adhesion of bacteria. Moreover, the R89BS coating was able to significantly interfere with biofilm formation and growth, depending on the specific microbial strains. An equivalent inhibitory effect was obtained for both the biomass and the cell metabolic activity of the *S. aureus* biofilm, whereas the metabolic activity was inhibited more than the biomass build-up of the *S. epidermidis* biofilm. This can be explained by the fact that the biomass of *S. epidermidis* ATCC^®^ 35984^TM^ is characterized by a prevalence of slime that may increase the amount of Crystal Violet used to quantify the biomass.

Overall, it is remarkable that the R89BS coating was able to guarantee a comprehensive anti-biofilm effect on both tested strains up to three days, while keeping a non-toxic effect on eukaryotic cells exposed to its eluate. These aspects make the R89BS coating a promising candidate for developing silicone medical devices with anti-biofilm properties.

## 4. Materials and Methods

### 4.1. Strains

The biosurfactant-producing strain *Pseudomonas aeruginosa* 89 was isolated from a patient with cystic fibrosis and stored at −80 °C in Tryptic Soy Broth (Biolife Italiana, Monza, Italy) with 25% glycerol. Biofilm producer strains of *Staphylococcus aureus* ATCC^®^ 6538^TM^ and *Staphylococcus epidermidis* ATCC^®^ 35984^TM^, stored at −80 °C in Brain Heart Infusion (Merck KGaA, Darmstadt, Germany) with 25% glycerol, were used in biofilm assays.

### 4.2. Biosurfactant Production

A loopful of *P. aeruginosa* 89, from a Tryptic Soy Agar (TSA) overnight culture, was added to 40 mL of Nutrient Broth II (Sifin Diagnostics GmbH, Berlin, Germany) and incubated for 4 h at 37 °C at 140 rpm. Afterward, 24 mL of this culture was inoculated in 1.2 L of Siegmund–Wagner medium (0.84 g of KH_2_PO_4_, 1.08 g of Na_2_HPO_4_, 2.40 g pf NaNO_3_, 0.48 g of MgSO_4_·7H_2_O, 0.12 g of CaCl_2_·2H_2_O, 2.4 mL of a trace element solution, (0.40 g of FeSO_4_·7H_2_O, 0.30 g of MnSO_4_·H_2_O, and 0.12 g of (NH_4_)6Mo_7_O_24_·4H_2_O in 200 mL of distilled water), 36 mL of glycerol, and 1161.60 mL of distilled water (pH adjusted to 6.7)) and incubated at 37 °C for five days at 120 rpm. At the end of the incubation period, the broth culture was centrifuged (Sorvall RC-5B Plus Superspeed Centrifuge, Fisher Scientific Italia, Milano, Italy) for 20 min at 6000 rpm. Free-cell supernatant was retrieved, acidified using 6 M H_2_SO_4_ at pH 2.2, and stored overnight at 4 °C. Eventually, biosurfactant R89 (R89BS) was extracted three times with ethyl acetate (Merck KGaA, Darmstadt, Germany), and the organic phase was evaporated to dryness under vacuum conditions.

### 4.3. Chemical Characterization of Biosurfactant R89

An aliquot of the crude biosurfactant extract was dissolved in methanol to obtain a 1 mg/mL stock solution. Freshly prepared working solutions were made by diluting the stock solution with methanol to 10 µg/mL solutions.

Mass spectrometry analyses were done on an LCQ DECA XP Plus (Thermo Finnigan, San Jose, CA, USA) IonTrap mass instrument equipped with an ESI source. Samples (10 µg/mL solutions) were injected with a syringe at 5 mL/min flow rate. Source voltage and capillary voltage were at 5.0 kV and 18 V in negative mode. The capillary temperature was maintained at 300 °C, and nitrogen was used as nebulizing gas at 50 arbitrary units. Data were acquired in negative MS total ion scan mode (mass scan range *m*/*z* 100–1000) and MS/MS product ion scan mode with normalized collision energy (nce%) optimized for each precursor ion selected: *m/z* 475, 531, and 649, 27%, 503, 25%, 677, 29%, and 621, 31%.

### 4.4. Antimicrobial Test on Planktonic Cells

The susceptibility of *Staphylococcus* planktonic cells to biosurfactant R89 was determined in 96-well microtiter plates using the broth dilution method according to Wiegand et al. [56] with some modifications. The tested concentration of R89BS ranged from 0.03 to 0.25 mg/mL. Bacterial suspensions (5 × 10^5^ CFU/mL) were inoculated in Mueller-Hinton Broth (Sifin Diagnostics GmbH, Berlin, Germany) in the presence of different R89BS concentrations. In control wells, the inoculum was mixed with an equal volume of PBS. Plates were incubated at 37 °C for 16–20 h. Blank wells were also included. The minimal inhibitory concentration (MIC) of R89BS was determined as the lowest concentration of the molecule that inhibited the growth of the tested strains as observed with the unaided eye. The observed vancomycin MICs (*S. aureus* MIC 1 μg/mL; *S. epidermidis* MIC 2 μg/mL), in line with the EUCAST International MIC distribution Reference Database (https://mic.eucast.org/Eucast2/SearchController/), were used as a reference during the evaluation of the antimicrobial activity of R89BS.

Finally, absorbance (A) at 595 nm was measured for each well (Ultramark Microplate Imaging System, Bio-Rad Laboratories S.r.l., Segrate, Italy), data were normalized with respect to the blank value, and the percentage of bacterial growth reduction was calculated as indicated in Section 4.10.

Assays were conducted in triplicate and repeated on three different days.

### 4.5. Preparation of Medical-Grade Silicone Substrate

Discs of 10 mm in diameter and 1.5 mm in thickness were cut from medical-grade silicone sheets (TECNOEXTR s.r.l, Palazzolo sull’Oglio, Italy). Cleaning and sterilization of silicone elastomeric discs (SEDs) were performed as indicated in Ceresa et al. [57]. Briefly, SEDs were washed with a 1.4% (*v*/*v*) RBS^TM^ 50 solution (Sigma-Aldrich), sonicated for 5 min, and rinsed twice in Milli-Q water. SEDs were then dipped in methanol (99%), sonicated for 5 min, and rinsed as above. Afterward, the material was sterilized by autoclave (121 °C, 15 min), dried at 37 °C for 20 h, and moved aseptically into 24-well plates.

### 4.6. Disruption of Pre-Formed Biofilms

For each strain, appropriate inoculum density, media, and growth conditions for biofilm formation were used. In particular, for *S. aureus*, glucose was added as a biofilm formation inducer, whereas, for *S. epidermidis*, cultivation under shaking was selected to promote slime production.

Bacterial suspensions were prepared at a cell density of 1 × 10^7^ CFU/mL in Tryptic Soy Broth (TSB) for *S. epidermidis* or TBS supplemented with 1% glucose (TSBG) for *S. aureus*. Experiments were conducted on a 24-well polystyrene plate, placing one SED per well. Each SED was submerged in 1 mL of inoculum and incubated at 37 °C for 24 h at 160 rpm for *S. epidermidis* or in static conditions for *S. aureus*. SEDs with biofilms formed on the surface were then carefully transferred into new plates, treated with different concentrations of R89BS (0.06–2 mg/mL), and incubated for a further 24 h. The dislodging activity of R89BS was evaluated by means of the MTT reduction assay detailed below. Assays were conducted in triplicate and repeated on two different days.

### 4.7. Silicone Surface Coating and Physicochemical Characterization

BS coating via physical adsorption was obtained by immersing silicone coupons or SEDs in 1 mL of a 2 mg/mL R89BS solution (R89BS-coated SEDs) for 24 h at 37 °C at 180 rpm. Control coupons or SEDs were immersed in 1 mL of PBS only. The R89BS solution was aspirated; then, SEDs were transferred to new plates and dried under a laminar flow cabinet (Heraeus Herasafe HS18, Kendro Laboratory Products, Hanau, Germany).

Contact angle was measured by the Whilelmy method using a Cahn DCA 322 microbalance (Cahn Scientific, Irvine, CA, USA) and Milli-Q^®^ (MerkMillipore, ‎Burlington, MA, USA) water at room temperature as the average value from five samples.

In order to detect compositional surface modifications, attenuated total reflectance Fourier-transform infrared spectroscopy (ATR-FTIR, FT-IR Spectrometer Frontier, PerkinElmer, Milan, Italy) was performed on control SEDs and R89BS-coated SED samples using a Perkin Elmer Spectrum. One spectrometer was equipped with a Diamant crystal. IR spectra were collected in the 4000 and 600 cm^−1^ wavenumber range at a resolution of 4 cm^−1^ and averaged over 32 scans. Spectra were analyzed by Spectrum software (PerkinElmer, Milan, Italy).

### 4.8. Anti-Biofilm Assays

Bacterial suspensions were prepared at a cell density of 1 × 10^7^ CFU/mL in TSB for *S. epidermidis* or TBS + 1% glucose (TSBG) for *S. aureus*. R89BS-coated SEDs and control SEDs were submerged in 1 mL of bacterial suspension and incubated at 37 °C at 160 rpm (*S. epidermidis*) or in static conditions (*S. aureus*) for 24, 48, and 72 h. Every 24 h, SEDs were transferred onto new plates containing fresh media. At the end of the incubation period, the growth medium was removed, and SEDs were gently washed twice with PBS to remove non-adherent cells.

Samples were prepared in triplicate and repeated on two different days.

#### 4.8.1. Biofilm Quantification

The anti-biofilm effect of R89BS-coated SEDs was quantitatively determined by Crystal Violet (CV) staining (biofilm biomass) and an MTT (3-(4,5-dimethylthiazolyl-2-yl)-2,5-diphenyltetrazolium bromide) reduction assay (cell metabolic activity).

For the evaluation of total biomass, biofilms were dried and stained with 1 mL of CV solution (0.2%) for 10 min. Blank SEDs (without biofilm) were also included. SEDs were washed with distilled water to remove dye excess and air-dried. The CV amount of each SED was determined spectrophotometrically after solubilization in 1 mL of acetic acid (33% in water).

For the determination of cell metabolic activity, biofilms were dipped in 1 mL of 0.3% MTT solution supplemented with 0.01% glucose and 1 μM menadione. After 30 min of incubation at 37 °C in static conditions, formazan crystals into biofilms were dissolved with 1 mL of a dimethyl sulfoxide (DMSO)/0.1 M glycine buffer (pH 10.2) solution (7:1).

A_570_ of the solutions was measured (Victor3V^TM^, Perkin Elmer, Milan, Italy). Data were normalized, and the percentage of biofilm reduction was calculated with the formula explained in Section 4.10.

#### 4.8.2. Scanning Electron Microscopy Analysis of Bacterial Biofilms

A quantitative and qualitative analysis of biofilms was also performed by scanning electron microscopy (SEM) adapting the protocol described in Ceresa et al. [58]. Quantitative SEM analysis was conducted with a XL30 ESEM FEG (Fei, Eindhoven, The Netherland) scanning electron microscope at a 15-kV beam voltage in low-vacuum mode, using a vapor pressure of 1.4 Torr to avoid excessive dehydration of the biofilm and the need for applying a conductive coating. A set of nine different fields of view for each SED was obtained by collecting the backscattered electrons signal at a magnification of 60×, thus guaranteeing the observation of a total area of 29 mm^2^ at the silicone disc center. To distinguish between biofilm-covered surface and exposed silicone, high-resolution digital images (1936 × 1452 pixels) were acquired and then thresholded using a semi-automated routine implemented in ImageJ (National Institutes of Health, Bethesda, MD, USA) according to the protocol used by Bressan et al. [59]. Percentage area of the silicone disc covered by bacterial biofilms (BA%) was calculated as explained in Section 4.10. Assays were carried out in triplicate.

Qualitative characterization of biofilm micromorphology and architecture was investigated using a Quanta 200F FEG (Fei, Eindhoven, The Netherlands ) scanning electron microscope in high-vacuum mode after coating the samples with a 10-nm layer of gold with a sputter coater (Emitech K500X, Quorum Technologies, Laughton, UK). Images at 5000× magnification were acquired to detect fine morphological details of cells and the extracellular matrix by collecting the secondary electron signal with a primary beam energy of 2 keV. Possible artefacts due to the sample preparation process were considered according to indications provided by Hrubanova et al. [60].

### 4.9. R89BS and R89BS-Coated SED Cytotoxicity

The potential cytotoxicity of R89BS and R89BS-coated SEDs was evaluated by a lactate dehydrogenase (LDH) assay (ISO 10993) (TOX7 In Vitro Toxicology Assay Kit, Sigma-Aldrich, Darmstadt, Germany), using normal lung fibroblasts (MRC5), according to TOX7 operative procedures [61]. The cell line was maintained in modified Eagle’s medium (MEM) supplemented with 10% fetal calf serum (FCS), l-glutamine (2 mM), sodium pyruvate (1mM), 1% non-essential amino acids, and 1% antibiotics at 37 °C, 5% CO_2_, and 95% relative humidity. R89BS-coated SEDs were immerged in fresh cell culture medium at 37 °C for 24 h in static or dynamic conditions (obtained by orbital shaking at 1 Hz to improve biosurfactant removal from the surface). At the same time, cells were seeded in 96-well tissue culture plates and cultured in standard medium until about 70% confluence (24 h). Afterward, the growth medium was removed and replaced with the medium containing R89BS solutions at different concentrations (1.0, 0.5, 0.4, 0.3, 0.2, 0.1 mg/mL) or the conditioned surface-contacting medium (200 µL/well). The cytotoxic effect was measured on the basis of the amount of LDH released by cells after 48 h of exposure. The positive control for cytotoxicity constituted fully lysate cells (0.5% Triton X), while cells in reduced medium without surfactant constituted the negative control. LDH level was evaluated by light absorbance at 490 nm (Tecan Spark 10 M). Assays were carried out in quintuplicate.

### 4.10. Data Analysis and Statistics

In tables and graphs, data were reported as means ± standard deviation. Confidence intervals (95%) of the ratio between values obtained from the control SEDs (Ctrl) and R89BS-coated SEDs (R89BS) were calculated from the corresponding confidence intervals for log_Ctrl_ − log_R89BS_.

The inhibition percentages (INHIBIT%) of bacterial growth, biofilm formation, and biofilm disruption were calculated with the following formula:

(1) $$\mathrm{INHIBIT}\%=\left(1-\frac{T}{\mathrm{Ctrl}}\right)\times 100$$

where T is the absorbance value of treated samples, and Ctrl is the absorbance value of the control. 

The percentage of the SED area covered by biofilms (BA%) was calculated as follows:

(2) $$\mathrm{BA}\%=\left(\frac{\mathrm{dark}\;\mathrm{pixel}}{\mathrm{total}\;\mathrm{pixel}\;\mathrm{number}}\right)\times 100$$

where dark pixels represent the biofilm-covered surface, and total pixel number represents the total SED surface.

The percentage of cytotoxicity (CYTOTOX%) was calculated as follows:

(3) $$\mathrm{CYTOTOX}\%=\left(\frac{A_{R89\mathrm{BS}}-A_{\mathrm{neg}.\mathrm{Ctrl}}}{A_{\mathrm{pos}.\mathrm{Ctrl}}-A_{\mathrm{neg}.\mathrm{Ctrl}}}\right)\times 100$$

where A_R89BS_ is the absorbance of samples treated with R89BS; A_pos.Ctrl_ is the absorbance of positive control (0.5% Triton X), and A_neg.Ctrl_ is the absorbance of negative control (growth medium).

Statistical analyses and graphics were elaborated by means of the statistical program R, 3.5.2. (R Development Core Team, http://www.R-project.org). One-way ANOVA followed by Tukey post hoc test was performed to study the effect of the different concentrations of R89BS (considered as factor variables) on pre-formed biofilms of each *Staphylococcus* strain and to evaluate the significance of data in the LDH cytotoxicity assay in comparison to positive and negative controls. Two-way ANOVA and a *t*-test were performed to investigate the effect of R89BS-coated SEDs on each *Staphylococcus* strain biofilm formation. Results were considered to be statistically significant when *p* < 5 × 10^−2^.

## Figures and Tables

**Table 1:** Effect of R89 biosurfactant (R89BS) on *Staphylococcus* spp. planktonic cells. Results are represented as means and standard deviations (SD). Minimal inhibitory concentration (MIC) values are provided, as observed with the unaided eye. OD—optical density.

Test Strain	R89BS Concentration (mg/mL)	OD at 595 nm (*n* = 6) (mean ± SD)
*S. aureus* ATCC^®^ 6538^TM^	Ctrl	0.847 ± 0.013
	0.03	0.772 ± 0.017
	0.06	0.004 ± 0.006
	0.12	0.001 ± 0.006
	0.25	0.001 ± 0.006
*S. epidermidis* ATCC^®^ 35984^TM^	Ctrl	0.918 ± 0.039
	0.03	0.768 ± 0.065
	0.06	0.630 ± 0.062
	0.12	0.001 ± 0.004
	0.25	0.001 ± 0.007

**Table 2:** The 95% confidence intervals and *p*-values calculated for *Staphylococcus* spp. biofilm total biomass and cell metabolic activity in pre-coating assays. Ctrl—control.

Time (h)	Strain	Total Biomass		Cell Metabolic Activity	
		95% Confidence Interval for the Ratio Ctrl/R89BS	*p-*Value (*t*-Test)	95% Confidence Interval for the Ratio Ctrl/R89BS	*p*-Value (*t*-Test)
24	*S. aureus*	(4.33, 5.35)	1.50 × 10^−7^	(3.65, 5.35)	4.77 × 10^−^^6^
	*S. epidermidis*	(2.65, 3.04)	2.22 × 10^−8^	(3.65, 4.36)	1.09 × 10^−^^7^
48	*S. aureus*	(4.50, 5.10)	2.28 × 10^−10^	(3.95, 4.89)	3.27 × 10^−^^8^
	*S. epidermidis*	(2.30, 2.54)	3.63 × 10^−9^	(2.83, 3.10)	4.30 × 10^−^^11^
72	*S. aureus*	(3.13, 3.49)	6.36 × 10^−^^9^	(3.29, 3.95)	4.22 × 10^−10^
	*S. epidermidis*	(1.95, 2.08)	6.43 × 10^−^^11^	(2.56, 2.88)	4.08 × 10^−10^

**Table 3:** Inhibition percentages of *Staphylococcus* spp. biofilm formation detected by CV and MTT assays. Relative inhibitions in the percentage of biofilm-covered surface obtained from SEM analysis are also reported.

Time (h)	Strain	Biomass (CV)	Metabolic Activity (MTT)	Surface Coverage (SEM)
24	*S. aureus*	79.2%	77.1%	69.9%
	*S. epidermidis*	64.6%	74.9%	40.3%
48	*S. aureus*	79.1%	77.2%	81.4%
	*S. epidermidis*	58.4%	66.2%	51.1%
72	*S. aureus*	69.6%	72.3%	78.0%
	*S. epidermidis*	50.2%	63.1%	52.8%

**Table 4:** Mean absorbance values (A_570_) ± standard deviation obtained in the MTT assays for evaluation of live cells in the supernatants of R89BS-coated silicone elastomeric discs (SEDs) and control SEDs at the different time points.

Time (h)	*S. aureus*		*S. epidermidis*	
	Control SEDs	R89BS-Coated SEDs	Control SEDs	R89BS-Coated SEDs
24 h	3.06 ± 0.05	9.09 ± 0.19	18.74 ± 1.88	31.24 ± 4.05
48 h	7.73 ± 0.63	9.79 ± 0.62	37.90 ± 5.12	45.73 ± 3.38
72 h	8.47 ± 1.20	11.42 ± 1.58	51.09 ± 5.46	60.94 ± 2.86
